# Effect of the Simultaneous Addition of Polycaprolactone and Carbon Nanotubes on the Mechanical, Electrical, and Adhesive Properties of Epoxy Resins Cured with Ionic Liquids

**DOI:** 10.3390/polym15071607

**Published:** 2023-03-23

**Authors:** Lidia Orduna, Itziar Otaegi, Nora Aranburu, Gonzalo Guerrica-Echevarría

**Affiliations:** POLYMAT and Department of Advanced Polymers and Materials: Physics, Chemistry and Technology, Faculty of Chemistry, University of the Basque Country (UPV/EHU), Paseo Manuel de Lardizabal 3, 20018 Donostia-San Sebastian, Spain

**Keywords:** epoxy resin, ionic liquid, nanocomposite, mechanical properties, electrical conductivity, lap shear strength

## Abstract

Electrically-conductive epoxy nanocomposites (NCs) with improved mechanical and adhesive properties were achieved through the combined addition of poly(ε-caprolactone) (PCL) and carbon nanotubes (CNTs). Three different ionic liquids (ILs) were used as dual role agents, i.e., as both curing and dispersing agents. Regardless of the IL used, the epoxy/PCL matrix of the NCs showed a single-phase behaviour and similar glass transition (T_g_) and crosslinking density (ν_e_) values to the unfilled epoxy/PCL/IL systems. Although the CNTs were more poorly dispersed in the epoxy/PCL/CNT/IL NCs than in the reference epoxy/CNT/IL NCs, which led to slightly lower electrical conductivity values, the epoxy/PCL/CNT/IL NCs were still semiconductive. Their low-strain mechanical properties (i.e., flexural modulus and flexural strength) were similar or better than those of the reference epoxy/IL systems and their high-strain mechanical properties (i.e., deformation at break and impact strength) were significantly better. In addition, the positive effects of the PCL and the CNTs on the adhesive properties of the epoxy/IL system were combined. The substitution of ILs for traditional amine-based curing agents and biodegradable PCL for part of the epoxy resin represents an important advance on the road towards greater sustainability.

## 1. Introduction

Epoxy resins are widely used thanks to their excellent mechanical, adhesive, chemical, and thermal properties. In addition, depending on the application, they can be modified to enhance these properties and/or compensate for any shortcomings. Their innate brittleness, for example, makes them unsuitable for use whenever toughness is required, so, traditionally, to overcome this, they have been blended with rubbers [1,2], oils [3,4,5], thermoplastics [6,7,8,9,10,11,12,13,14], or even carbonaceous nanofillers [15].

Morphology is known to be one of the main factors that define the final properties of polymer blends. Indeed, in the literature on epoxy resins blended with thermoplastics, different morphologies have been reported, depending on the nature of the thermoplastic, its molecular weight [14,16,17] or concentration [14], or on the hardener [7,10,16,18] or curing conditions used [10,19,20,21]. Poly(ε-caprolactone) (PCL), for instance, has been widely used for improving the high-strain mechanical properties of epoxy resins [10,11,12,13,18]. Anhydrides, when used as curing agents in epoxy/PCL blends, have been known to give rise to immiscible blends [22,23], while amines generally produce miscible blends [13,18,24] thanks to the hydrogen bonding that takes place with the ester groups of the PCL. Elsewhere, other amines have produced contradictory results. For instance, 4,4′-diaminodiphenylsulfone (DDS) has led to phase-separated blends [12,18,19,25].

Another way to improve the properties of neat epoxy resins and epoxy/thermoplastic systems is by adding nanofillers. Carbon nanotubes (CNTs) are widely used because by adding them to polymer matrices, electrically-conductive materials with enhanced mechanical, thermal, and/or adhesive properties can be obtained. However, in order to obtain optimal results, the interaction between both components must be optimal, and this is why the functionalization of CNTs, either covalent or noncovalent, has attracted so much interest among researchers [26,27,28] and has often been employed in epoxy/CNT systems [29,30,31,32]. A wide variety of different functionalization methods can be found in the literature, varying from the introduction of amine groups, epoxy groups, ozone, oxidation and so on. Moreover, in thermoset systems, the preparation method of the NCs is deemed crucial to achieving the required CNT dispersion level. Different preparation methods can be found in the bibliography, from magnetic stirring [33], a combination of sonication and stirring [34]—similar to that used in the present work—milling [35,36], extrusion [37] and even the spraying of a previously sonicated and stirred CNT solution [38].

In ternary epoxy/PCL/CNT nanocomposites, the PCL increases the viscosity of the blend which, in turn, improves the dispersion of the CNTs [39,40] so that a smaller amount is necessary for the nanocomposites to become semiconductive.

Finally, one of the main environmental concerns associated with epoxy resins is the use of toxic, volatile hardeners. This is why imidazolium- [41,42,43,44,45,46] and phosphonium- [45,46,47,48,49,50,51,52] based ionic liquids (ILs) have been proposed as more sustainable epoxy curing agents. ILs are salts that melt at temperatures below 100 °C and have excellent properties: they are comparatively stable, are ionically conductive, and have a low vapour pressure. In addition to being non-volatile, smaller amounts of ILs are needed to cure epoxy resins because they act as initiators rather than as co-monomers [47]. They have also been reported as effective curing agents in several epoxy/thermoplastic systems, such as epoxy/poly(2,6-dimethyl-1,4-phenylen ether) (PPE) [53], epoxy/polyetherimide (PEI) [54], and epoxy/poly(ε-caprolactone) [45], which we included in our previous work. Furthermore, ILs have been used as dispersing agents in CNT-filled thermoplastic [55,56,57] and thermoset [58,59,60,61] NCs.

To summarise, epoxy/PCL and epoxy/CNT systems have already been widely studied, as is the case, to a lesser extent, of ternary epoxy/PCL/CNT NCs. However, no work has investigated, to the best of our knowledge, the advantages—in terms of mechanical, electrical, and adhesive properties—of simultaneously adding a PCL toughener and CNTs to IL-cured epoxy systems. Thus, in this work, and based on previous results, three ILs were selected for use as curing agents in ternary epoxy/PCL/CNT NCs, and the morphology as well as the thermal, mechanical, electrical, and adhesive properties were characterised and compared with those of the reference epoxy/CNT systems.

## 2. Materials and Methods

### 2.1. Materials

The diglycidyl ether of bisphenol A (DGEBA) epoxy resin used in this work had an epoxy equivalent weight of 186 g and was purchased from Nazza, Eurotex (Madrid, Spain). The poly(ε-caprolactone) (PCL) used was CAPA6800 (Ingevity, North Charleston, SC, USA), with a molecular weight of 80,000 g/mol. The multi-walled carbon nanotubes were Nanocyl NC7000 (L = 1.5 µm, D = 9.5 nm, 250–300 m^2^/g surface area, 90% purity) supplied by Nanocyl (Sambreville, Belgium). Three different ionic liquids were used as curing agents for the epoxy resin: trihexyltetradecylphosphonium dicyanamide (IL-P-DCA) from IoLiTec-Ionic Liquid Technologies GmbH (Heilbronn, Germany), and 1-ethyl-3-methylimidazolium dicyanamide (IL-I-DCA) and trihexyltetradecylphosphonium bis(2,4,4-trimethylpentyl) phosphinate (IL-P-TMPP) from Sigma Aldrich, St. Louis, MI, USA.

### 2.2. Preparation of Samples

Based on our previous results, the PCL content selected was 20 wt.% [45] with 0.2 wt.% CNTs [62]. Before preparing the samples, the DGEBA was degassed in a vacuum oven at 80 °C for 1 h. The epoxy/PCL/CNT/IL NCs and the reference epoxy/IL, epoxy/PCL/IL, and epoxy/CNT/IL systems were prepared as follows (see flowcharts in Figure S1):

Epoxy/PCL/CNT/IL: The DGEBA and the PCL were mechanically stirred at 100 °C until a homogenous mixture was obtained (≈2 h). After degassing the mixture, the CNTs were added and the resulting NC was mechanically mixed at 2000 rpm. It was subsequently ultrasonicated in a Hielscher UP400s at an amplitude of 100% for 20 min. Next, the IL was added and the blend was stirred at 100 °C until a homogenous mixture was obtained. Finally, the mixtures were either poured into moulds or between substrates, and the corresponding curing protocol was applied (Table 1).

Epoxy/IL: The DGEBA and IL were mechanically mixed at 50 °C for 5 min. Then, the mixtures were either poured into silicon moulds or between substrates and the corresponding curing protocol was applied (Table 1).

Epoxy/PCL/IL: The DGEBA and the PCL were mechanically mixed at 100 °C until a homogenous mixture was obtained (≈2 h). After degassing the mixtures, the IL was added and the blend was stirred at 100 °C until a homogenous mixture was obtained. Finally, the mixtures were poured into moulds or between substrates and the corresponding curing protocol was applied (Table 1).

Epoxy/CNT/IL: First, the epoxy resin and the CNTs were mixed and mechanically stirred at 2000 rpm. Next, the mixtures were ultrasonicated in a Hielscher UP400s at an amplitude of 100% for 20 min. After that, the IL was added and the mixtures were stirred at 50 °C for 5 min. Finally, they were poured into moulds or between substrates, and cured using the curing protocol shown in Table 1.

### 2.3. Characterization

#### 2.3.1. Phase Behaviour

The glass transition temperature (T_g_) and the crosslinking density (*ν_e_*) were determined by dynamic mechanical analysis (DMA) in a TA Q800 viscoelastometer (New Castle, DE, USA) in single cantilever bending mode. A temperature range of −100 °C to 250 °C was used at a heating rate of 4 °C/min. The frequency and amplitude used were 1 Hz and 15 μm, respectively. Equation (1) was used to calculate the crosslinking density [63]:

(1) $$\nu_{e}=\frac{E_{r}}{3RT_{r}},$$

where *R* is the ideal gas constant (8.314 J/mol·K), *E_r_* is the storage modulus in the rubbery state, and *T_r_* is the temperature at which *E_r_* was taken (245 °C).

Differential scanning calorimetry (DSC) was performed in a Perkin Elmer DSC-7 calorimeter (Waltham, MA, USA) calibrated with an indium standard. The samples, which were taken from the cured specimens, were heated from 30 °C to 250 °C at 20 °C/min under a nitrogen atmosphere.

#### 2.3.2. Microstructure

Scanning electron microscopy (SEM) was used to study the morphology. A Hitachi TM3030Plus microscope (Tokyo, Japan) equipped with a secondary electron detector and with an accelerating voltage of 15 kV was employed. Cryofractured cross sections were analysed, and the samples were gold-coated prior to observation.

#### 2.3.3. Nanostructure

Transmission electron microscopy (TEM) was used to study the dispersion level of the CNTs. Samples were cut at a 45° angle using a Leica EM UCG ultramicrotome equipped with a diamond blade. A Tecnai G2 20 Twin microscope (Hillsboro, OR, USA) was used at an accelerating voltage of 200 kV.

#### 2.3.4. Electrical Properties

Electrical conductivity measurements were carried out using a digital Keithley 6487 picoammeter (Cleveland, OH, USA). For the measurement, 2-mm-thick samples measuring 70 mm in diameter were used. A voltage of 1 V was applied, and intensity values were read after 1 min of electrification time. The electrical conductivity (*σ*) was calculated using Equation (2):

(2) $$\sigma =\frac{1}{\rho }=\frac{thickness\;\left(\mathrm{cm}\right)\times I}{22.9\times V}\;\;\;\left(S/\mathrm{cm}\right),$$

where 22.9 is a geometrical factor, *V* is the applied voltage, *ρ* is the resistivity, and *I* is the intensity reading on the picoammeter.

#### 2.3.5. Mechanical Properties

The mechanical properties were determined by performing bending tests in an Instron 5569 universal testing machine (Norwood, MA, USA) equipped with a three-point bending device (crosshead speed: 2 mm/min; span: 64 mm). Samples with the ISO 178 standard measurements (80 × 10 × 4 mm^3^) were tested. A minimum of 5 specimens were tested for each composition.

The flexural modulus (*E_f_*), flexural strength (*σ_F_*), and deformation at break (*ε_F_*) were calculated using Equations (3), (4), and (5), respectively:

(3) $$E_{f}=\frac{FL^{3}}{4sbh^{3}},$$

(4) $$\sigma_{F}=\frac{3F_{max}L}{2bh^{2}},$$

(5) $$\varepsilon_{F}\;\left(\%\right)=\frac{6sh}{L^{2}}\times 100,$$

where *F* and *F_max_* are the load and maximum load, respectively, *L* is the span, *b* is the width of the specimen, *h* is the thickness, and *s* is the deflection.

The impact resistance of the systems was measured by Charpy impact tests, using a Ceast 6548/000 impact tester (Norwood, MA, USA). Notched specimens (depth: 2.54 mm, radius: 0.25 mm) were used. At least 8 samples were tested for each reported value.

#### 2.3.6. Adhesive Properties

The adhesive properties were evaluated by means of lap shear strength tests. An Instron 5569 universal testing machine was used with a crosshead rate of 1 mm/min. Aluminium alloy 2021-T351 sheets (100 × 25 × 1.6 mm^3^) were used as substrates (Rocholl GmbH, Eschelbronn, Germany). The tests were performed according to the ASTM D-1002 standard (adhesion area: 12.5 × 25 mm^2^). The lap shear strength was calculated by dividing the maximum force in the force-displacement curve by the adhesion area. At least 10 specimens were tested for each reported value.

## 3. Results

### 3.1. Phase Structure

**Figure 1:**
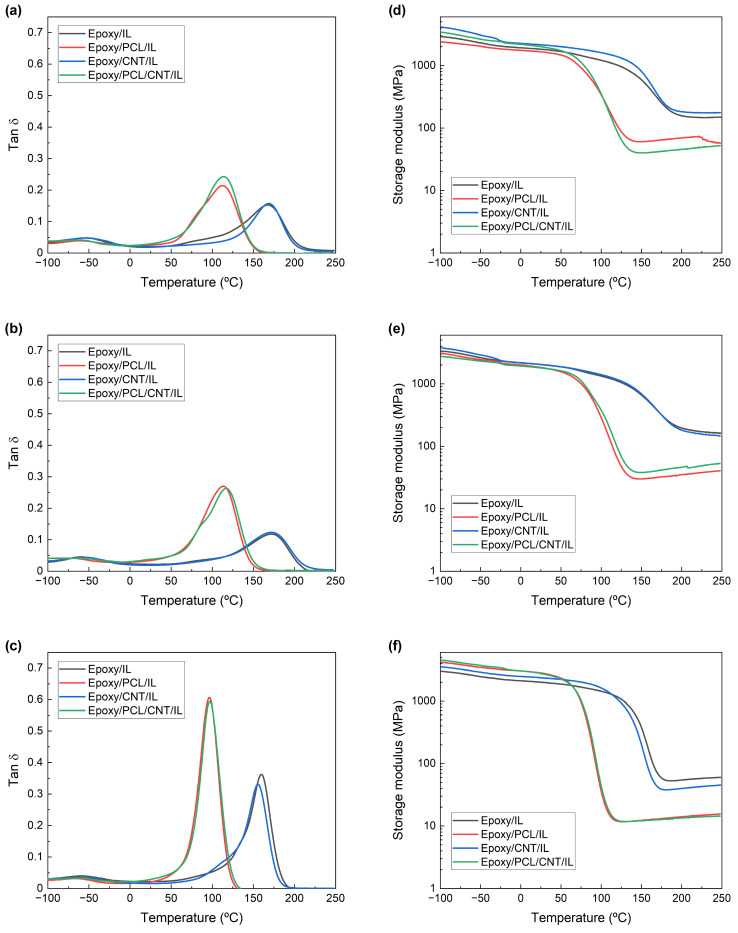
The tanδ (**a**–**c**) and storage modulus (**d**–**f**) vs. temperature curves of the epoxy/IL, epoxy/PCL/IL, epoxy/CNT/IL, and epoxy/PCL/CNT/IL systems cured with IL-P-TMPP (**a**,**d**), IL-P-DCA (**b**,**e**), and IL-I-DCA (**c**,**f**).

Figure 1 shows the tanδ and storage modulus vs. temperature curves of the epoxy/PCL/CNT NCs cured with the three ILs. The curves corresponding to the reference epoxy/IL, epoxy/PCL/IL, and epoxy/CNT/IL systems are also shown. The T_g_ and ν_e_ obtained from these curves are summarised in Table 2.

All the systems showed a single α transition peak corresponding to the T_g_, regardless of the IL employed. The addition of the PCL to the epoxy resin caused a drop in the T_g_. Moreover, a lower crosslinking density was observed in the epoxy/PCL/IL systems. These results point to the miscibility of the epoxy resin with the PCL [10,18,45]. Regarding the effect of adding CNTs to the epoxy/IL system, neither the T_g_ nor the ν_e_ changed significantly in the epoxy/CNT/IL NCs compared to the unfilled epoxy resin. The literature is not conclusive about the effect of adding nanofillers in general—or CNTs in particular—on the phase behaviour of epoxy/thermoplastic blends. While some authors have reported no effect when different nanofillers were added to epoxy/thermoplastic systems [64,65], others have reported a drop in the T_g_ when CNTs were added, attributing it to the miscibilisation of the thermoplastic caused by the CNTs [66,67].

The T_g_ and ν_e_ of both the epoxy/PCL/CNT/IL NCs and the epoxy/PCL/IL systems were similar, with the CNTs causing no noticeable effect. Thus, regardless of the IL used, the advantages of the PCL and the CNTs were combined by adding them at the same time. Furthermore, just like the unfilled epoxy/PCL/IL systems, the epoxy/PCL/CNT/IL NCs showed a single T_g_, pointing to a single-phase, homogenous, miscible system, proving that the combined presence of the PCL and CNTs did not modify the single-phase behaviour of the epoxy/PCL/IL systems.

The possible crystallization of the PCL, which would be indicative of phase separation, was investigated by DSC in the epoxy/PCL/CNT NCs cured with the three ILs. No evidence of an endothermic peak related to the melting of PCL appeared, regardless of the IL employed. This is additional evidence of the miscibility of the epoxy resin and the PCL in the NCs [45], as ascertained by DMTA.

### 3.2. Microstructure and Nanostructure

The morphology of the epoxy/PCL/CNT/IL NCs was analysed by SEM. No sign of phase separation was observed in any of the NCs, regardless of the IL employed, which is consistent with the results in the previous section. Similar results were also obtained in our previous study [45] for unfilled epoxy/PCL/IL systems, indicating that the presence of the CNTs did not modify the phase structure.

**Figure 2:**
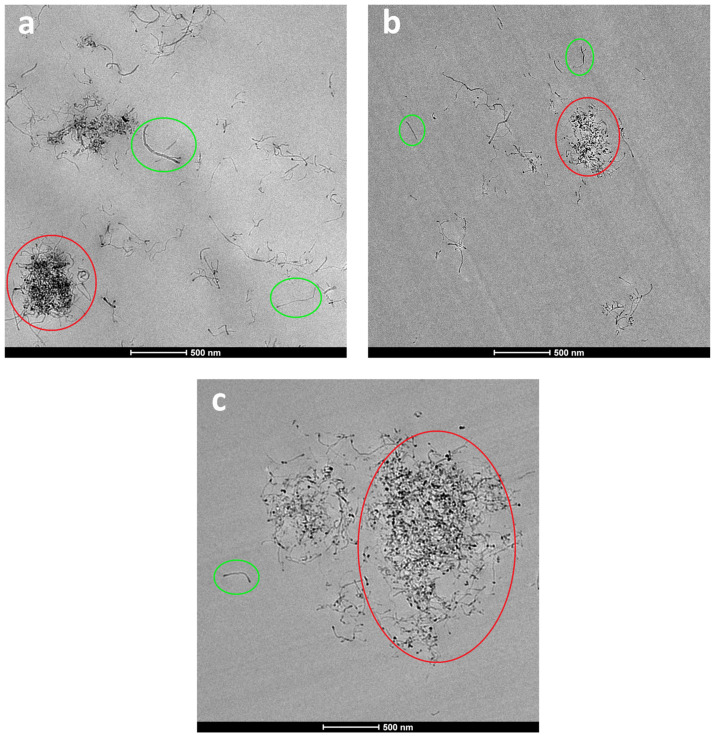
TEM micrographs of the epoxy/PCL/CNT NCs cured with (**a**) IL-P-TMPP, (**b**) IL-P-DCA, and (**c**) IL-I-DCA.

Figure 2 shows the TEM micrographs of the epoxy/PCL/CNT NCs cured with the three ILs. Regardless of the IL used, areas with both individual, well-dispersed CNTs (green circles) and aggregates (red circles) were observed in the three systems. Compared to the corresponding PCL-free reference systems (i.e., the epoxy/CNT/IL NCs) [62], the aggregates were bigger and greater in number in the epoxy/PCL/CNT/IL NCs, indicating poorer dispersion of the CNTs in the presence of PCL. This is probably because the epoxy/PCL blend was more viscous than the epoxy resin. The preparation technique used in this study is based on low shear forces (mechanical stirring and ultrasonication) and the greater viscosity hindered the dispersion of the CNTs during the preparation of the NCs.

**Figure 3:**
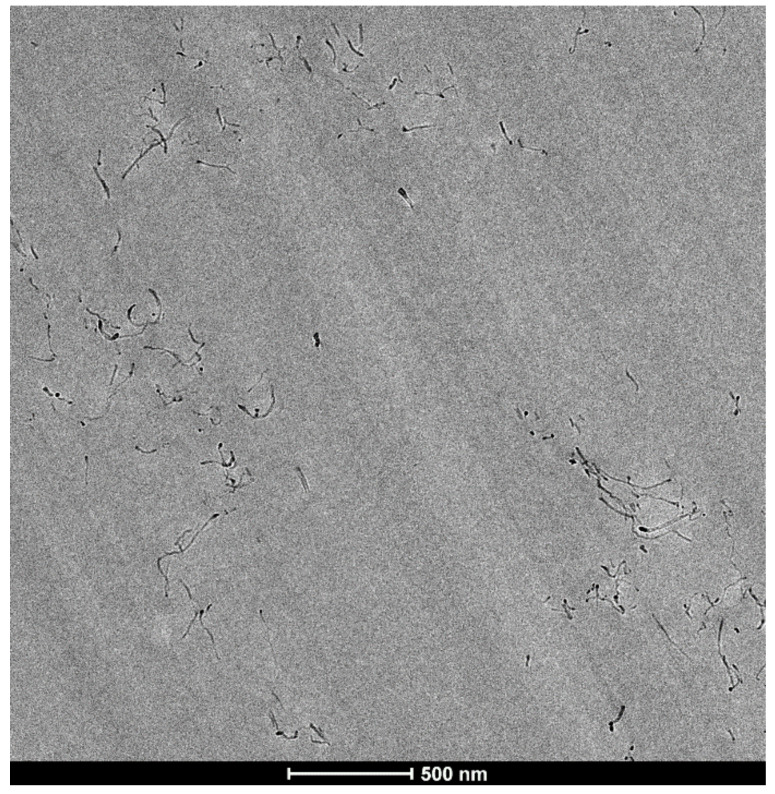
TEM micrograph of the IL-P-DCA-cured epoxy/PCL/CNT NCs.

A systematic observation and general evaluation of the surfaces was carried out to qualitatively compare the degree of dispersion of the CNTs in the NCs cured with the three different ILs. The best dispersion (largest area of well-dispersed CNTs, with fewer and smaller aggregates) was observed for the NC cured with IL-P-DCA, followed by the IL-P-TMPP-cured NC. In contrast, the IL-I-DCA-cured NC showed the poorest dispersion. Figure 3 shows a micrograph of the well-dispersed epoxy/PCL/CNT/IL-P-DCA blend where an area of very well-dispersed CNTs can be seen.

As previously mentioned, the viscosity of the epoxy/PCL mixture is one of the key factors that affects the dispersion of the CNTs in the NCs. During the preparation of the samples, the addition of the ILs to the epoxy/PCL mixture reduced the viscosity, partially compensating for the increase caused by the PCL. The epoxy/PCL/IL blend that contained IL-P-DCA was the least viscous, followed by the IL-P-TMPP NC. In contrast, the addition of the IL-I-DCA to the epoxy/PCL barely reduced the viscosity of the mixture. This is consistent with the dispersion observed by TEM, and was further confirmed by the electrical properties discussed below.

### 3.3. Electrical Properties

**Figure 4:**
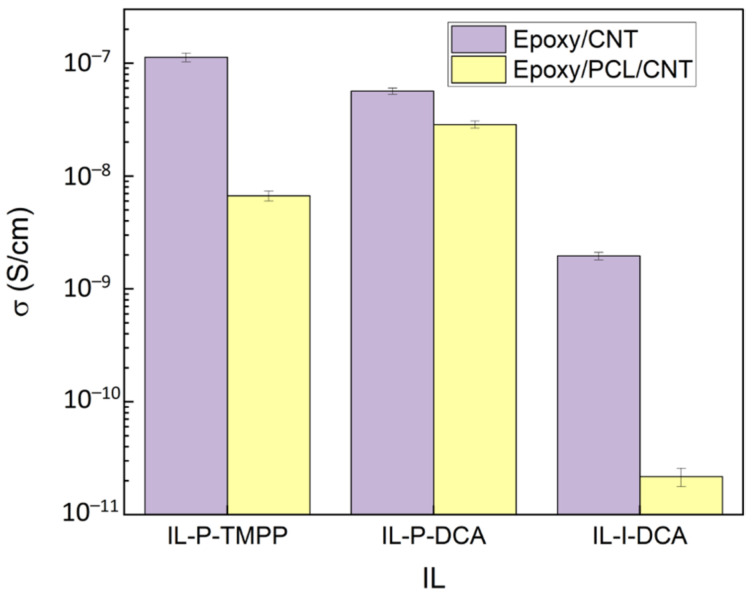
Electrical conductivity of the epoxy/PCL/CNT NCs and the reference epoxy/CNT NCs cured with the three different ILs.

Figure 4 shows the electrical conductivity of the epoxy/PCL/CNT/IL NCs and the reference epoxy/CNT/IL NCs. As can be seen, the electrical conductivity of the NCs decreased when the PCL was added, regardless of the IL used. When the NCs with the different ILs are compared, the drop in conductivity of the IL-P-DCA NC was the lowest compared to the epoxy/CNT/IL system, while that of the IL-I-DCA NC was the highest. These results are perfectly consistent with the nanostructure discussed in the previous section. In the literature, however, the opposite effect has also been observed when PCL was added: the increase it caused in viscosity led to better-dispersed CNTs and, consequently, better electrical properties [39,40]. This is probably because the experimental procedure used for preparing the NCs was different. In any case, it is noteworthy that, in this study, despite the drop in electrical conductivity observed when the PCL was added to the epoxy/CNT/IL NCs, the three epoxy/PCL/CNT/IL NCs were still above the percolation threshold composition and were semiconductive, in contrast with the insulating nature of the pure epoxy/IL compositions (10^−14^–10^−15^ S/cm) [62]. When compared to the data from the literature [68,69,70], the electrical conductivity values reached fell within the ranges found for epoxy-based NCs filled with low CNT contents (10^−9^–10^−3^ S/cm for NCs containing 0.2–0.3 wt% CNT).

### 3.4. Mechanical Properties

**Figure 5:**
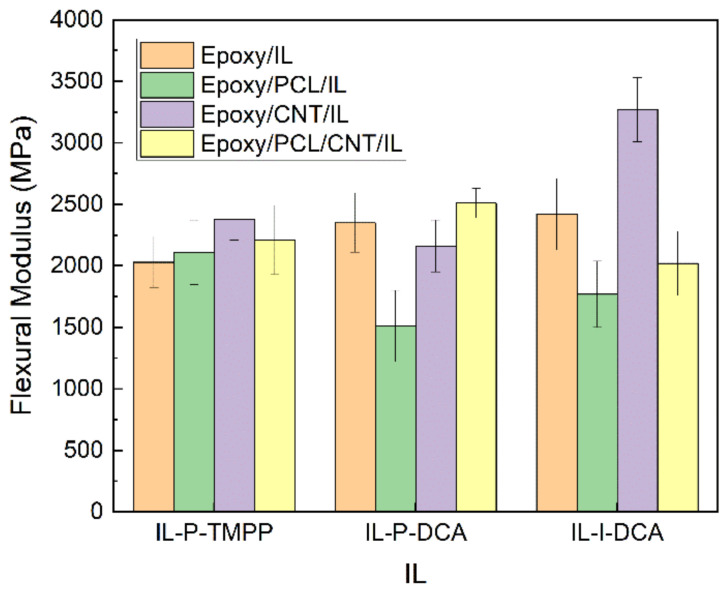
The flexural modulus of the epoxy/PCL/CNT NCs cured with the three different ILs, as well as that of the reference epoxy/IL, epoxy/PCL/IL and epoxy/CNT/IL systems.

**Figure 6:**
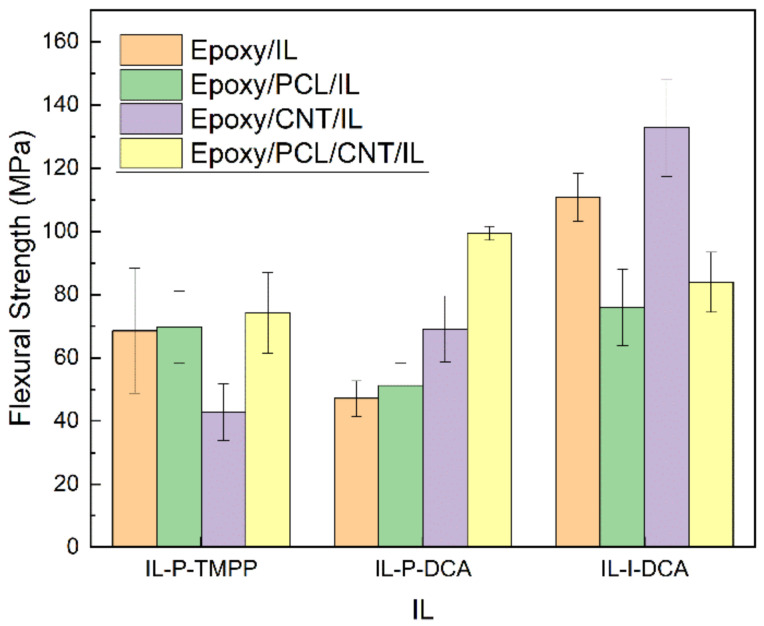
The flexural strength of the epoxy/PCL/CNT NCs cured with the three different ILs, and of the reference epoxy/IL, epoxy/PCL/IL and epoxy/CNT/IL systems.

**Figure 7:**
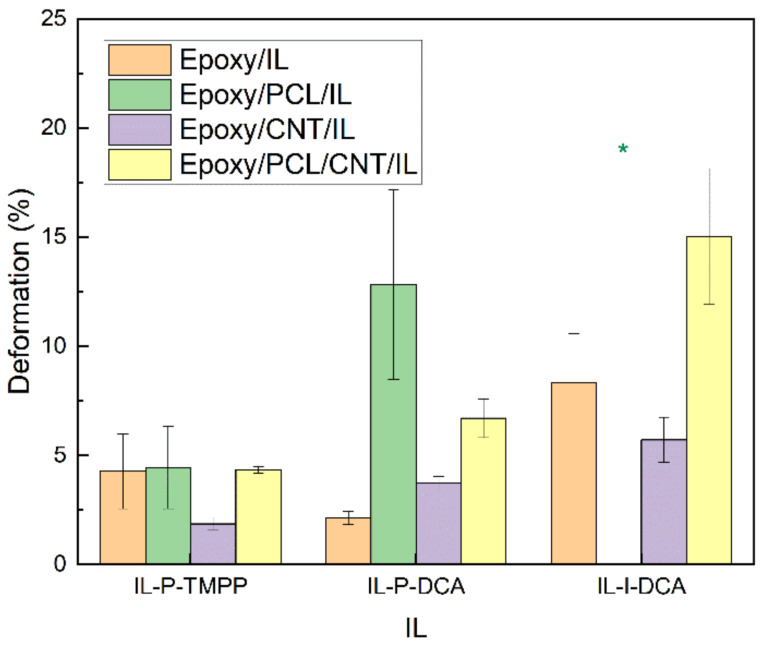
The deformation at break of the epoxy/PCL/CNT NCs cured with the three different ILs, as well as that of the reference epoxy/IL, epoxy/PCL/IL and epoxy/CNT/IL systems. * The specimen slipped and did not break.

**Figure 8:**
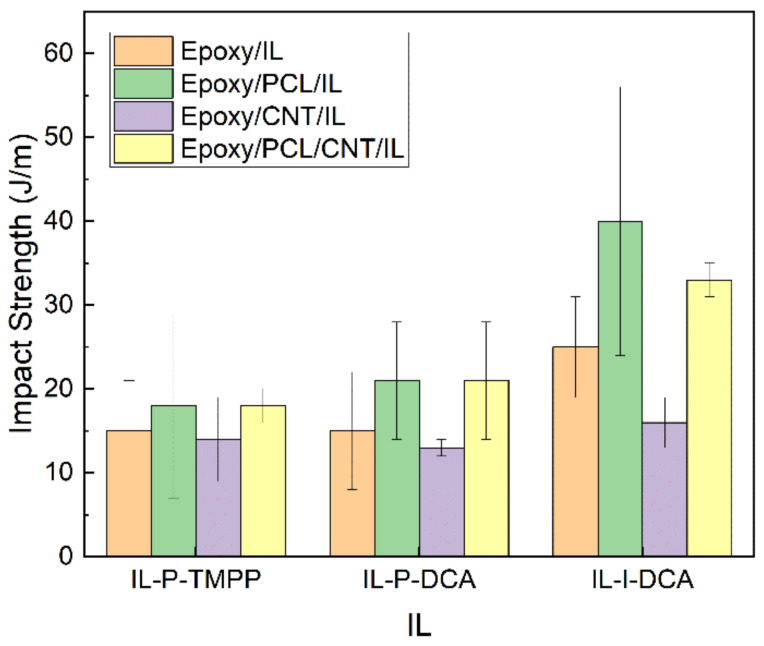
The impact strength of the epoxy/PCL/CNT NCs cured with the three different ILs, as well as that of the reference epoxy/IL, epoxy/PCL/IL and epoxy/CNT/IL systems.

The mechanical performance of the epoxy/PCL/CNT/IL NCs and the reference epoxy/IL, epoxy/PCL/IL, and epoxy/PCL/CNT/IL blends was tested using bending and impact tests. Figure 5, Figure 6, Figure 7, and Figure 8, respectively, show the flexural modulus, flexural strength, ductility, and impact strength of systems cured with the three different ILs.

Regarding the low-strain mechanical properties (i.e., flexural modulus and strength), it is clear from Figure 5 and Figure 6 that the simultaneous addition of PCL and CNTs to the epoxy resin resulted in an improvement in the low-strain mechanical properties of the NCs cured with IL-P-TMPP and IL-P-DCA. The flexural modulus and strength of the epoxy/PCL/CNT NC cured with IL-P-TMPP increased by 9% and 8%, respectively, with respect to the reference epoxy resin while the flexural modulus and strength of the IL-P-DCA-cured NC increased by 7% and 110%, respectively. The IL-I-DCA-cured NC was the only one in which the flexural modulus and strength dropped when the PCL and CNT were added to the epoxy resin. The results of the IL-P-TMPP and IL-P-DCA blends are particularly noteworthy because although thermoplastic elastomers are normally added to improve high-strain mechanical properties, they usually have a negative impact on low-strain mechanical properties. The positive effect in this study may be due to the reinforcing efficiency of the CNTs [71,72], which offsets the negative impact of the PCL on the low-strain mechanical properties.

As for high-strain mechanical properties, the ductility and impact strength of the epoxy/PCL/CNT/IL NCs and the reference systems are shown in Figure 7 and Figure 8, respectively. As is common [66], the presence of CNTs had a negative effect on the high-strain mechanical properties, as reflected in the lower values of the epoxy/CNT/IL and the epoxy/PCL/CNT/IL NCs compared to the respective unfilled reference epoxy/IL and epoxy/PCL/IL systems. The decreases in the epoxy/PCL/CNT/IL system were not so dramatic, however, as the differences fell mostly within the standard deviation of the measurement. Most remarkably, the ductility and impact strength of all the epoxy/PCL/CNT/IL NCs increased compared to the reference epoxy/IL systems. For example, the ductility of the epoxy/PCL/CNT/IL NCs with the IL-P-DCA and the IL-I-DCA were 219% and 80% greater, respectively, than that of the corresponding reference epoxy/IL systems, while the impact strength of the epoxy/PCL/CNT/IL-I-DCA NC increased by 32%.

In conclusion, the epoxy/PCL/CNT NCs cured with the different ILs presented an excellent balance of mechanical properties, showing similar or superior low-strain properties to the reference epoxy/IL and epoxy/PCL/IL systems, and did not in any way affect the positive impact of PCL on the high-strain mechanical properties.

### 3.5. Adhesive Properties

**Figure 9:**
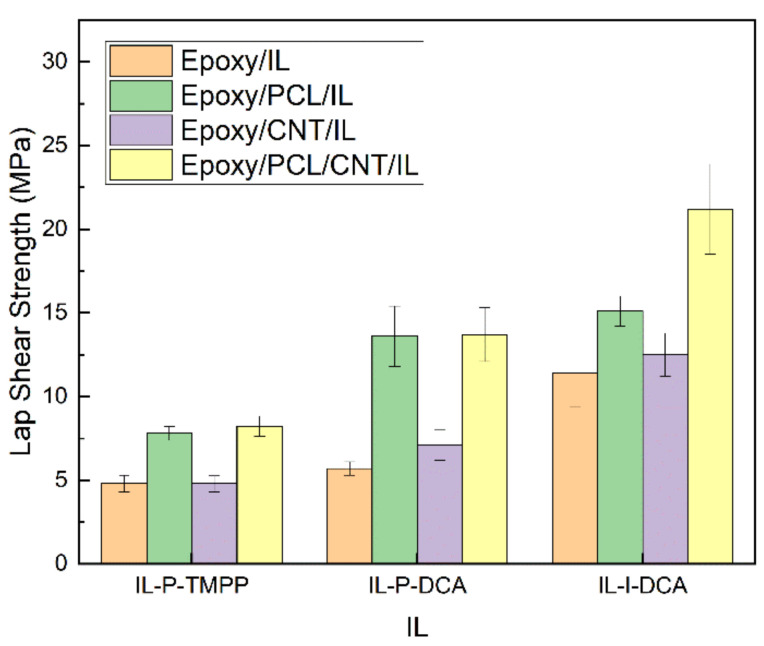
The lap shear strength of the epoxy/PCL/CNT NCs cured with the three different ILs, as well as that of the reference epoxy/IL, epoxy/PCL/IL and epoxy/CNT/IL systems.

The adhesive properties of the epoxy systems in the study were measured by lap shear tests. Figure 9 shows the results.

As reported in one of our previous studies [45] and reflected in Figure 9 here, the adhesive properties of an epoxy/IL system can be significantly improved by adding PCL, regardless of the IL used. This is due to the decrease in the crosslinking density as observed by DMTA and also to the increase in toughness caused by the presence of PCL, as discussed in the previous section [73,74,75]. The addition of CNTs also had a positive effect on the lap shear strength, as reported in the literature [62,76,77,78], albeit to a lesser degree. In the epoxy/PCL/CNT/IL NCs, the combined effect of the two components was both beneficial and additive, and, in some cases, even synergistic (e.g., in the IL-I-DCA-cured NCs). Regardless of the IL used, the epoxy/PCL/CNT/IL NCs had the best lap-shear strength values. Compared to the corresponding reference epoxy/IL systems, the lap-shear strength of the NC cured with IL-P-TMPP improved by 73%, while that of the IL-P-DCA and IL-I-DCA NCs improved by 140% and 86%, respectively.

## 4. Conclusions

The effect of the simultaneous addition of PCL and CNTs to an epoxy resin cured with three different ionic liquids was studied. The miscibility of the epoxy resin and the PCL remained constant in the epoxy/PCL/CNT/IL NCs. While the addition of PCL led to a decrease in both the T_g_ and the crosslinking density of the reference epoxy/IL systems, the effect of adding the CNTs was negligible.

Regardless of the IL used, the resulting epoxy/PCL/CNT/IL NCs had an excellent balance of mechanical properties. The low-strain properties (i.e., flexural modulus and strength) improved in most cases with respect to the reference epoxy/IL and epoxy/PCL/IL systems, while the high-strain mechanical properties (ductility and impact strength) improved or did not disimprove significantly compared to the epoxy/IL and epoxy/PCL/IL systems, respectively. Moreover, the low-strain mechanical properties were similar or better than the reference epoxy/CNT/IL systems, while the high-strain mechanical properties improved considerably.

The combined addition of PCL and CNTs also led to an outstanding improvement in the adhesive properties; the lap shear strength of the epoxy/PCL/CNT/IL NCs was better than that of any of the reference blends.

Finally, despite the decrease in electrical conductivity caused by the presence of PCL and the poorer dispersion of the CNTs caused by the increase in viscosity, the epoxy/PCL/CNT/IL NCs were still considered semiconductive.

Thus, the simultaneous addition of PCL and CNTs to epoxy systems cured with different ILs resulted in semiconductive materials with enhanced mechanical and adhesive properties. Because ionic liquids are non-volatile hardeners and can be used in lower concentrations than traditional amine-based curing agents, and because part of the DGEBA epoxy resin was replaced with biodegradable PCL, the blends in the present work represent an important contribution to the production of more sustainable materials.

The optimization of the CNT and PCL contents in the NCs achieved in this study and their resulting enhanced balance of properties could pave the way for these NCs being used in advanced applications such as electronics packaging, wind turbine blades, structural adhesives, and sporting goods, and could also provide an interesting area for future research.

## Figures and Tables

**Table 1:** Curing protocol used with each IL.

Curing Agent	Curing Protocol
IL-P-TMPP	2 h 80 °C/2 h 120 °C/1 h 150 °C/1 h 170 °C
IL-P-DCA	2 h 120 °C/2 h 140 °C/1 h 170 °C
IL-I-DCA	2 h 110 °C/1 h 140 °C/1 h 170 °C

**Table 2:** The glass transition temperatures and crosslinking densities of the epoxy/PCL/CNT/IL NCs and the reference epoxy/IL, epoxy/PCL/IL, and epoxy/CNT/IL systems, cured with the three different ILs.

System	T_g_ (°C)	ν_e_ (mol/m^3^)
IL-P-TMPP		
Epoxy/IL	168	11,509
Epoxy/PCL/IL	112	4487
Epoxy/CNT/IL	169	13,594
Epoxy/PCL/CNT/IL	115	4012
IL-P-DCA		
Epoxy/IL	172	12,616
Epoxy/PCL/IL	113	3117
Epoxy/CNT/IL	173	11,437
Epoxy/PCL/CNT/IL	117	4079
IL-I-DCA		
Epoxy/IL	160	4625
Epoxy/PCL/IL	96	1197
Epoxy/CNT/IL	155	3599
Epoxy/PCL/CNT/IL	97	1116

## Data Availability

Not applicable.

## Supplementary Materials

The following supporting information can be downloaded at: https://www.mdpi.com/article/10.3390/polym15071607/s1, Figure S1: Flowchart of the preparation process of the epoxy/PCL/CNT/IL NCs, as well as that of the reference epoxy/IL, epoxy/PCL/IL, and epoxy/CNT/IL systems.
