# Supplementary material for: Effect of the Simultaneous Addition of Polycaprolactone and Carbon Nanotubes on the Mechanical, Electrical, and Adhesive Properties of Epoxy Resins Cured with Ionic Liquids
Source: Polymers (Basel). 2023 Mar 23;15(7):1607. doi: 10.3390/polym15071607 (PMC10097337; doi:10.3390/polym15071607)
Supplement: Supplementary file 1 [file polymers-15-01607-s001.zip › polymers-2254381-supplementary.pdf]

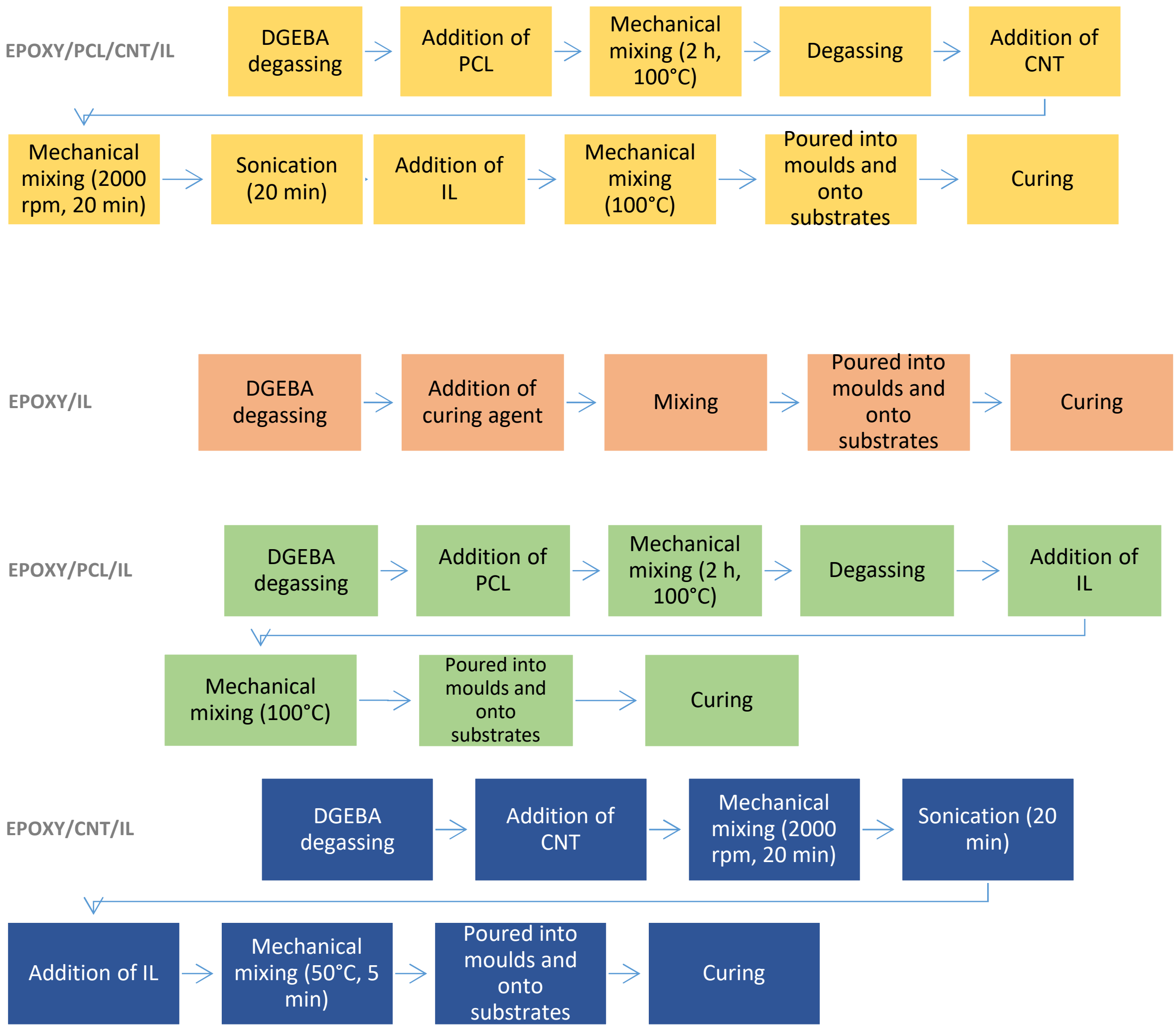

Figure S1. Flowchart of the preparation process of the epoxy/PCL/CNT/IL NCs, as well as that of the reference epoxy/IL, epoxy/PCL/IL, and epoxy/CNT/IL systems.
